# Cognitive Remediation for Individuals with Psychosis in a Supported Education Setting: A Pilot Study

**DOI:** 10.1155/2012/715176

**Published:** 2012-05-15

**Authors:** Sean A. Kidd, Jaswant Kaur Bajwa, Kwame J. McKenzie, Rohan Ganguli, Bahar Haji Khamneh

**Affiliations:** ^1^Schizophrenia Program, Psychology Service Centre for Addiction and Mental Health, Department of Psychiatry, University of Toronto, 250 College Street, Suite 738, Toronto, ON, Canada M5T 1R8; ^2^Redirection Through Education, George Brown College, 200 King Street East, Toronto, ON, Canada M5T 2T9; ^3^Social Equity and Health Research, CAMH, University of Toronto, 455 Spadina Avenue, Suite 300, Toronto, ON, Canada M5S 2G8; ^4^Centre for Addiction and Mental Health, University of Toronto, 901 King Street West, Suite 500, Box 13, Toronto, ON, Canada M5V 3H5; ^5^Schizophrenia Program, Centre for Addiction and Mental Health, University of Toronto, 250 College Street, Suite 738, Toronto, ON, Canada M5T 1R8

## Abstract

Cognitive remediation (CR) is a treatment approach that is being increasingly examined as a means through which the cognitive impacts of schizophrenia might be ameliorated. While CR has demonstrated good outcomes when paired with supported employment, little is known regarding how it might be integrated within supported education contexts. In this study CR was examined in a supported education context with 16 individuals with psychosis. The findings indicated that CR aligned well with the academic curriculum with very low attrition, was found useful by students, and showed similar pre-post differences on cognitive measures as those found in previous work.

## 1. Introduction

A large body of research has demonstrated the presence of significant cognitive challenges among persons with schizophrenia with demonstrated abilities, on average, 1-2 standard deviations lower than the general population in areas of problem solving, attention, and memory [1]. These cognitive impacts of schizophrenia tend to be persistent and are minimally impacted by psychotropic medication [2]. Their impact is felt in numerous domains including interpersonal functioning, independent living, occupational functioning, and skill acquisition in psychiatric rehabilitation [3, 4]. Cognitive challenges are particularly salient in work and school settings, which require people to multitask, sustain attention, and recall material presented in a range of formats. Given the central role of education and employment in the recovery of many persons with severe mental illness [5], there is a pressing need to identify effective treatments that address the challenges that can limit progress in these functional domains.

Cognitive remediation is a promising approach for addressing the cognitive impacts of schizophrenia. Cognitive remediation (CR) refers to interventions in which cognitive tasks are practiced to improve attention, memory, and problem solving abilities. Randomized controlled trials have consistently shown beneficial impacts on both cognitive and psychosocial functioning [6]. Key findings include (i) moderate effect sizes for improvements in attention, memory, and problem solving [6, 7], (ii) more modest impacts on psychosocial functioning with better outcomes observed when CR is paired with supported employment [2, 6, 8], (iii) a relatively low impact on psychosis symptomatology, though self-esteem has been found to improve [6, 9], and (iv) maintenance of improvement for periods of up to 2 years after intervention [8]. Examination of nonspecific effects has indicated that CR leads to significant benefit over and above tasks that capture nonspecific factors (e.g., computer skills training) [10].

Building from criticisms of the narrow scope of earlier CR studies which looked only at pre and post measures of cognitive functioning and their questionable association with “real world” outcomes [11], there has been an increasing emphasis upon pairing CR with evidence-based psychiatric rehabilitation interventions. In the research literature, this pairing has primarily been undertaken in the context of employment-based interventions with evidence suggesting that CR can enhance their effectiveness [6, 8, 12, 13]. However, we have been unable to locate any report of the impact of cognitive remediation training when paired with a supported education intervention for persons with mental illness or any other population. Supported education settings incorporate assistance, preparation, and ongoing support to adults with psychiatric disabilities who wish to pursue postsecondary education or training, with the first example developed in the 1980s at the Boston University Center for Psychiatric Rehabilitation [14]. Outcome studies of supported education programs have found better enrolment in postsecondary education and longer terms of competitive employment [15]. While widely used, however, the evidence base for supported education has lagged behind other similar interventions (e.g., supported employment) due to a lack of randomized trials and variability between education programs.

Given the importance of supported education in psychiatric rehabilitation, and the close association between supported education and supported employment suggesting transferability, we conducted a feasibility study to examine the impact of cognitive remediation when paired with a supported education intervention.

## 2. Methods

### 2.1. Cognitive Remediation and Supported Education

This study employed a pre-post measure design that examined changes in cognitive functioning and symptomatology over the course of an academic term among individuals with psychosis who took part in a CR intervention while enrolled in a supported education program. To facilitate the comparability of the results, the measures used were adapted from those of McGurk and colleagues [16]. This evaluation also included a qualitative component to evaluate participant impressions about the intervention.

The study took place in Toronto, Ontario, at the Redirection Through Education (RTE) program which is situated within a mainstream college setting—George Brown College. Note that in the Canadian context the term “college” (as opposed to “university”) refers to a postsecondary program with a greater emphasis on training for skilled trades and professions such as Chef and Dental Hygienist. Redirection Through Education is a supported education program for adults, 19 years of age and older, who are facing challenges with mental health and/or addictions issues. The RTE program provides students with the opportunity to assess and improve their academic skills to facilitate entry into employment and nonsupported training and education settings. Students enroll in credit courses such as College English, Computer Skills, Speaking with Confidence, Strategies for Student Success, and Psychology of Human Relations. These courses can lead to eligibility and/or exemptions in postsecondary programs depending on the grades achieved. Other noncredit courses include Foundational Skills in English. Students take 8-9 mandatory classes per semester along with any electives they might choose with work and volunteer placements occurring in semesters 2 and 3 of the 3-semester program. In addition to classes, students are provided with individual counselors to assist in their developing effective study skills, goal setting, and coping with the stresses of an academic environment. RTE students also engage in vocational assessment and exploration to help determine their interests and strengths and to address areas of challenge. For further details about George Brown College and RTE go to: http://www.georgebrown.ca. While there is some variability from one academic term to the next, approximately 30% of RTE students have psychosis. The program typically has between 120 and 240 students enrolled, most of whom are young adults. The overarching goal of this program is to help students explore valued nonillness identities, build confidence, and reengage with their communities.

The CR intervention was integrated within the course structure of RTE and took place in college classrooms. The intervention had two components that were completed over the course of 10 weeks within the academic term. A total of 20-, 45-minute computer-based cognitive exercise sessions were held twice a week using COGPACK (COGPACK, version 6.0, Marker Software, Ladenburg, Germany, http://www.cogpack.de/). This computer program facilitates practice across a broad range of cognitive functions, including attention and concentration, psychomotor speed, learning and memory, and executive functioning. The protocol for the COGPACK exercises was manualized with a general progression from easier to more difficult tasks and through cognitive domains in the order described above. Generally, two cognitive exercises were practiced in each session with their receiving encouragement and suggestions about strategies for improving performance. There was, however, flexibility in the protocol allowing for individualized instruction and support. While all participants completed exercises in all cognitive domains, some were more readily able to progress to more difficult levels than others. Additionally, while there was a general progression through functional domains across sessions, there was some variation. For example, a difficult problem solving task might be accompanied by a less demanding attention task which has more of a readily engaged video game format. They all received performance scores on their accuracy and speed after completing each exercise, which were recorded in the computer and used to reinforce them for progress on their performance. COGPACK sessions were cofacilitated by a Clinical Psychologist and an MA level Psychology graduate student and were held in groups of 4-5 participants to facilitate individualized feedback.

In addition to computer exercises, participants took part in 10 weekly group discussion sessions (approximately 60 minutes in duration) attended by all 16 participants. Topics in the group sessopms included the role of cognitive factors in academic performance, the development of compensatory strategies for dealing with challenges in academic settings (e.g., study strategies, means of addressing attention difficulties), and strategies for managing difficulties such as anxiety and psychosis symptoms in school settings (e.g., breathing exercises, and mindfulness). These sessions were cofacilitated by a Clinical Psychologist and an education specialist affiliated with RTE. Group sessions were also manualized, drawing from evidence-based practices wherever possible (e.g., CBT skills for managing anxiety) with computer and discussion sessions synchronized such that group discussions aligned with COGPACK tasks.

### 2.2. Measures

All of the quantitative measures used are commonly employed in CR studies and have excellent psychometric properties. Quantitative measures were administered immediately before and after the CR intervention. *Symptoms of psychosis* were assessed using the Positive and Negative Syndrome Scale (PANSS) [17], and *self-esteem* was assessed with the Rosenberg Self-Esteem Scale [18]. The Wide Range Achievement Test (WRAT-III) reading subtest [19] was used to *evaluate premorbid educational attainment*. *Psychomotor speed* was measured using the Trail Making test part A [20], and *short-term memory *was evaluated with the digit span subtest of the Weschler Adult Intelligence Scale-III [21]. *Verbal learning and memory* was assessed with the California Verbal Learning Test (CVLT) [22], and *executive functioning* was assessed with the Trail Making Test, Part B and the Wisconsin Card Sorting Test (WCST) [23]. *Sustained attention/vigilance* was examined using the Digit Vigilance Test [24]. Through a focus group at midpoint and brief individual interviews at the end of CR, a semistructured inquiry format was used to explore what aspects of CR participants found helpful and unhelpful including attention to the training, the groups, and the logistics of completing CR while attending school. Interviews were audiorecorded, transcribed verbatim, with a thematic analysis used to determine common themes across responses [25].

### 2.3. Participants

 Of the 30 RTE students with psychosis who registered for the summer term, 17 enrolled in cognitive remediation. Of those 17, 16 participants completed the CR program. These 16 participants were comprised of 12 males and 4 females with a mean age of 30 years (range 18–54) of whom 11 were white, 2 East Asian, and 3 of African Caribbean descent. All participants had been identified as having psychosis as per their chart diagnosis documented in referral information (11 Schizophrenia, 5 other psychoses), and all were taking psychotropic medication for psychosis. The average amount of time since first diagnosis was 9.1 years (range 1–28 years). Eleven participants lived with family with a range of living circumstances across the other participants. They had an average of 12.3 years of education (range 8–16 years) and reported that alcohol and substance use was minimal. Mean alcoholic beverages consumed in a typical week was 1.0 (range 0–8) and mean marijuana use per week was 0.69 (range 0–7) with all other substance use denied. The pre-CR WRAT Reading Subtest mean standard score was 68 with a range of 49 to 86. While the WRAT scores of some participants were somewhat low, it is of note that none had a chart diagnosis of an intellectual disability. It is possible, however, that reading difficulties may have led to additional challenges in the completion of some CR tasks. On average, participants attended 16.4/20 computer training sessions and 7.7/10 group meetings.

## 3. Results

Table 1 summarizes pre-post evaluation findings. Mean and standard deviation statistics are provided along with significance test findings using the Wilcoxon Signed Rank test. Effect size calculations were made using Cohen's *d* for repeated measures. Significant improvement was noted on the Trail Making B test, verbal learning on the CVLT, the time component of the Digit Vigilance Task, and on the general psychosis symptomatology measure, the PANSS. Moderate effect sizes, but not statistical significance (likely due to the low sample size), were also seen on the Digit Span backwards subtest, and in decreases in the number of errors on the WCST and Digit Vigilance tasks. 

With respect to qualitative commentary about CR, the themes that emerged through the content analysis revealed extremely positive feedback. Of the 16 participants, 12 said that they found both computer and group sessions very enjoyable and 13 described experiencing an improvement in cognitive functioning, particularly memory and concentration. Most described these improvements as having generalized to other areas of functioning, particularly in the school setting.

“*I enjoyed the computer games. I liked the challenge. It showed if I have a test that I have the techniques to retain it all and keep it in there. Especially the scenes tasks, it helped my thinking process and showed me how take in a lot of information…. I used to have a hard time trying to concentrate and focus on one person and the training showed me how to do that. For example in class, it helped me to learn how to keep my mind clear and be able to focus and listen to the teacher.*”

“*I remember you telling me to make a mental picture of things I needed to remember. When I did that, my memory improved by like 15–25% which was a really big deal for me because normally I was just writing stuff down and that was it.*”

They stated that they found it rewarding and confidence-building to see the progress they made on tasks. Five participants described specific benefits from the group meetings, including being able to share struggles with others in a similar position, and improvements in mood and anxiety.

“*I also like the discussion group where people share different ideas, different experiences on how to do well with the computer activities… what really grabbed me is when your colleague said the brain is like a muscle and the more you exercise it the more efficient it is. That really grabbed me and that sort of influenced me to really take the enhancement study seriously.*”

Only 3 participants stated that they were unsure of whether or not CR had been beneficial for them. None of the participants described having a negative impression, and none expressed serious reservations about having participated.

## 4. Discussion

 This study has demonstrated that Cognitive Remediation (CR) can be readily integrated within a supported education setting. The students enjoyed CR, and most found it very helpful in improving their concentration and memory and, generally, learn and practice strategies that can ameliorate the cognitive impacts of their psychosis. Outcome findings were promising, with improvements noted over the course of the academic term in learning, concentration, and some aspects of executive functioning. Improvements in psychosis symptomatology were also seen. While the degree to which these changes might be attributed to CR cannot be determined in this study due to a lack of a control group, the general pattern of improvement is consistent with the impact of CR as observed in controlled trials [6]. One exception was the lack of change observed in self-esteem which has been found in other CR trials [6, 9]. Generalizability to other settings is difficult to determine due at least in part to supported education models being much less standardized than interventions such as supported employment [15]. The RTE program does, however, have many of the components common to supported education settings in other jurisdictions [26]. Finally, these findings must be considered in light of possible practice effects with respect to use of the testing instruments and the potential influence of demand characteristics.

## 5. Conclusions

 There is a compelling need for the ongoing development and enhancement of strategies to improve the quality of life of individuals with schizophrenia. While, for many, psychotropic medications assist in the reduction of the positive symptoms of the illness such as delusions and hallucinations, the negative symptoms and cognitive impacts pose a tremendous challenge in the recovery process. These factors can impede progress in most areas of functioning and impact the effectiveness of psychiatric rehabilitation interventions including supported employment [27] and supported education. In RTE, for example, teaching staff routinely see less success in course and program completion among students with psychosis, particularly for individuals with schizophrenia. It is in this context that restorative strategies such as CR and compensatory strategies such as Cognitive Adaptation Training [28] have the potential, as an adjunct to other interventions, to improve the likelihood of attaining better outcomes.

The present study would seem to complement the inroads that other investigators have made in examining the manner in which CR can enhance the impact of supported employment [6, 8, 12, 13]. We have demonstrated that CR is readily embedded in a supported education program and would seem to look promising in regard to effectiveness—certainly to an extent that further investigation using randomized trial designs is warranted. Those trials will be crucial in determining if the impact of CR warrants an investment in its implementation in supported education settings. Engaging in an economic analysis will be critical to gaining the support of administrators in funding relatively resource-intensive CR interventions in increasingly resource-scarce contexts.

## Figures and Tables

**Table 1:** Pre-post evaluation of cognitive remediation.

Instrument	Pre mean (SD)	Post mean (SD)	*Z*	*P*	Effect size *d*
Digit span (WAIS-III)					
Forward	9.25 (2.27)	9.68 (2.52)	−0.84	.403	0.32
Backward	5.81 (1.76)	6.44 (1.86)	−1.68	.093	0.63
Trail making					
Part A	36.25 (14.41)	38.00 (19.22)	−0.70	.485	−0.19
Part B	109.00 (43.48)	87.25 (42.21)	−2.05	.041*	0.96
California verbal learning test					
Trial 1	5.06 (.93)	6.06 (1.44)	−2.32	.021*	1.04
Trial 1–4	28.00 (2.45)	29.93 (3.77)	−2.34	.019*	1.16
Long delay free recall	6.81 (1.56)	7.13 (1.75)	−0.93	.353	0.37
Wisconsin card sorting test					
% Perseverative errors	20.31 (14.52)	15.31 (8.59)	−1.34	.181	0.52
% Conceptual level responses	45.88 (24.75)	48.50 (25.16)	−0.37	.711	0.22
Total categories	2.13 (1.59)	2.38 (1.59)	−1.10	.271	0.38
Digit vigilance test					
Total time	478.75 (131.31)	434.00 (96.95)	−2.07	.023*	0.64
Total errors	7.67 (7.60)	5.33 (5.23)	−1.37	.172	0.65
Rosenberg self-esteem scale	17.50 (6.47)	17.75 (5.70)	−0.21	.838	0.13
PANSS (total score)	56.19 (6.76)	49.50 (8.73)	−2.28	.023*	0.91

*P < .05; *N*: 16; (effect size strength: 0.20: small; 0.50: moderate; ≥0.80: large; Cohen 1977 [29]).
